# AAV-ie-mediated UCP2 overexpression accelerates inner hair cell loss during aging in vivo

**DOI:** 10.1186/s10020-022-00552-y

**Published:** 2022-10-20

**Authors:** Chunli Zhao, Zijing Yang, Zhongrui Chen, Wenqi Liang, Shusheng Gong, Zhengde Du

**Affiliations:** 1 Department of Otolaryngology Head and Neck Surgery, Beijing Friendship Hospital, Capital Medical University, No. 95, Yong’an Road, Xicheng, Beijing, 100050 China; 2 Clinical Center for Hearing Loss, Capital Medical University, Beijing, 100050 China

**Keywords:** UCP2, ARHL, IHC, AMPKα, Mitochondrion, Apoptosis

## Abstract

**Background:**

Uncoupling protein 2 (UCP2), activated by excessive reactive oxygen species (ROS) in vivo, has the dual effect of reducing ROS to protect against oxidative stress and reducing ATP production to regulate cellular metabolism. Both the UCP2 and ROS are increased in cochleae in age-related hearing loss (ARHL). However, the role of UCP2 in sensory hair cells in ARHL remains unclear.

**Methods:**

Male C57BL/6 J mice were randomly assigned to an 8-week-old group (Group 1), a 16-week-old group (Group 2), a 16-week-old + adeno-associated virus-inner ear (AAV-ie) group (Group 3), and a 16-week-old + AAV-ie-UCP2 group (Group 4). Mice aged 8 weeks were administrated with AAV-ie-GFP or AAV-ie-UCP2 via posterior semicircular canal injection. Eight weeks after this viral intervention, hearing thresholds and wave-I amplitudes were tested by auditory brainstem response (ABR). Subsequently, the cochlear basilar membrane was dissected for investigation. The number of hair cells and inner hair cell (IHC) synapses, the level of ROS, and the expression of AMP-activated protein kinase α (AMPKα), were assessed by immunofluorescence staining. In addition, mitochondrial function was determined, and the expression of AMPKα and UCP2 proteins was further evaluated using western blotting.

**Results:**

Mice with early-onset ARHL exhibited enhanced oxidative stress and loss of outer hair cells and IHC synapses, while UCP2 overexpression aggravated hearing loss and cochlear pathophysiological changes in mice. UCP2 overexpression resulted in a notable decrease in the number of IHCs and IHC synapses, caused ATP depletion and excessive ROS generation, increased AMPKα protein levels, and promoted IHC apoptosis, especially in the apical and middle turns of the cochlea.

**Conclusion:**

Collectively, our data suggest that UCP2 overexpression may cause mitochondrial dysfunction via energy metabolism, which activates mitochondrion-dependent cellular apoptosis and leads to IHC loss, ultimately exacerbating ARHL.

## Introduction

Age-related hearing loss (ARHL), or presbycusis, is the third source of disabilities in older adults. It is related to multiple factors, such as noise exposure, ototoxic medication, diet, smoking, chronic conditions, resulting in poorer quality of life, cognitive impairment, social withdrawal, and even dementia (WHO 2021). The histological changes in ARHL comprise degeneration of peripheral and central auditory pathways, including inner hair cells (IHCs), outer hair cells (OHCs), the stria vascularis, spiral ganglion neurons (SGNs), the auditory cortex, etc. (Fetoni et al. 2011). The function of IHCs greatly relies on the healthy mitochondria which are considered as the powerhouses that generate adenosine triphosphate (ATP) via oxidative phosphorylation (OXPHOS) (Nunnari and Suomalainen 2012). Sufficient ATP is necessary for maintaining cell homeostasis, and fewer free radicals are emitted when ATP is produced in mitochondria. Many studies have concluded that mitochondrial dysfunction is an important cause of IHC damage in ARHL (Du et al. 2019; Fetoni et al. 2011; Guo et al. 2020; Wagner and Shin 2019; Yamasoba et al. 2013). According to the free radical theory of aging, ARHL is associated with oxidative stress (Beckman and Ames 1998; Kauppila et al. 2017). The aging cochlea shows excessive reactive oxygen species (ROS) production, which can damage mitochondrial function and thus cause hair cells injury, ribbon synapses loss, SGNs degeneration, cell apoptosis, ultimately causing hearing loss. Besides, in experimental animal models, several mechanisms, such as inflammation, mitochondrial dysfunction, glutamate toxicity, and calcium overload also contribute to ARHL.

Uncoupling proteins (UCPs), primarily located in the inner mitochondrial membrane, participate in regulating cellular ROS and ATP generation. UCPs uncouple OXPHOS through protons leak, resulting in decreased mitochondrial membrane potential (MMP) and ROS generation. Mitochondrial UCP2, a subset of the UCP family, is ubiquitously expressed in multiple cells and tissues, such as the pancreas, spleen, kidney, brain, and immune system, with expression also existing in the cochlea (Du et al. 2020; Krauss et al. 2005). UCP2 also has tissue-specific features. For example, UCP2 acts as a vital link between islet β-cell dysfunction and type 2 diabetes as a negative regulator of insulin secretion (Zhang et al. 2001). Moreover, research has shown that that increased ATP and insulin production are characteristic of UCP2-deficient mice. Interestingly, UCP2 has a neuroprotective role in regulating cellular stress (Bai et al. 2018; Dutra et al. 2018). Its overexpression contributes to an increased number of mitochondria and higher ATP levels, and thus, effectively inhibiting neuronal death in the hippocampus (Diano et al. 2003).

UCP2 has a dual effect on the regulation of mitochondrial function involved in the controlling oxidative stress and energy homeostasis. The hypothesis of 'uncoupling to survive' indicates that enhanced coupling results in much more oxygen expenditure but reduces ROS levels (Brand 2000). Several studies have shown that UCP2 is essential for protecting cells against mitochondrial oxidative damage by reducing ROS generation and suppressing ROS-induced cell death (Hu et al. 2019; Park et al. 2011; Teshima et al. 2003; Tian et al. 2012). However, UCP2 also adversely affects ATP generation, resulting in decreased ATP levels or even energy crises through its proton leak activity that may induce cell death (Shang et al. 2009). Therefore, UCP2 has contradictory roles in modulating cell death.

Given the biological functions and characteristics of UCP2, there may be a subtle line between cell protection and cell deterioration. We have previously reported that endogenous UCP2 expression is significantly elevated in the cochlea and auditory cortex of D‐galactose‐induced ARHL in vivo (Du et al. 2020, 2014). In addition, ROS increase with age in ARHL. However, the role of UCP2 in cochlear hair cells of ARHL remains unknown. To explore the mechanism of UCP2 on IHCs in ARHL, we applied an adeno-associated virus-inner ear (AAV-ie)-mediated delivery of UCP2 into the cochlea. We found that UCP2 overexpression resulted in much more severe hearing loss and deterioration of IHCs and mitochondrial function in mice with ARHL.

## Materials and methods

### Animals

All animal protocols were approved by the Institutional Animal Care and Use Committee of Capital Medical University, Beijing, China. Due to naturally carrying the *Ahl* gene, C57BL/6 J mice show high-frequency hearing loss at the age of 8 weeks, and thus are widely used for ARHL studies (Erway et al. 1993). All mice were randomly assigned four groups: (1) group 1, normal 8-week-old (8w) mice; (2) group 2, normal 16-week-old (16w) mice; (3) group 3, 16-week-old + AAV-ie-GFP mice (normal 8w mice were treated with AAV-ie empty vector via the posterior semicircular canal (PSC) injection, 8 weeks after which they were used for subsequent experiments); (4) group 4, 16-week-old + AAV-ie-UCP2 mice (normal 8w mice were treated with AAV-ie-UCP2 via the PSC, 8 weeks after which they were examined).

### AAV-ie vector

The AAV-ie vector carrying UCP2, or empty vector with a CAG promoter and enhanced green fluorescent protein (AAV-ie-CAG-UCP2-eGFP or AAV-ie-CAG-eGFP), were manufactured by PackGene Biotech (Guangzhou, China). AAV-ie-CAG-eGFP was as control vector.

### Surgery

The operation was performed as previously descripted in our laboratory (Guo et al. 2018; He et al. 2020). Briefly, mice were anesthetized with ketamine (100 mg/kg) and xylazine (10 mg/kg) by intraperitoneal injection (i.p.). The PSC on the left ear was exposed and dilled a tiny hole using a needle. The polyimide tube was gently inserted into the PSC through the hole. A total of 2 μL of AAV-ie was injected into each cochlea within 4 min. AAV-ie empty vector and AAV-ie-UCP2 were transfected within the cochlea of mice in the group 3 and group 4, respectively. After the virus injection, the hole in the PSC was rapidly blocked using a small piece of muscle. And the skin incision was sutured and disinfect with povidone.

### Auditory brainstem response (ABR) testing

The method of ABR testing has been described previously (Liang et al. 2021; Sergeyenko et al. 2013). Briefly, it was conducted in sound-proof room using BioSigRZ software (Tucker-Davis Technologies, USA). Mice were anesthetized with ketamine (100 mg/kg) and xylazine (10 mg/kg). The condition of the external auditory canal and the tympanic membrane (TM) was observed using an electric otoscope before testing. Mice with acute otitis externa (AOE) and otitis media were excluded. AOE is characterized by an inflamed external auditory canal (Wiegand et al. 2019); The main manifestation of otitis media is a red, swollen, or even perforated TM, and may also include purulent exudate or middle ear effusion (Sundgaard et al. 2021). ABR response was recorded by subcutaneous needle electrodes. The recording, the reference and the ground electrode were located in subcutaneous tissue of the vertex of the skull, the mastoid process of the tested ear and the mastoid process of contralateral ear, respectively. The electrical signals were amplified, filtered and averaged (1024 samples). The sound level was diminished in 5 dB steps from 90 dB SPL to the hearing threshold. The response was elicited in tone bursts at four frequencies of 4, 8, 16, and 32 kHz. The hearing threshold was determined as the lowest stimulus level at which a repeatable Wave II could be visually identified. The amplitude of ABR waves was the difference between the peak and the subsequent trough (Sergeyenko et al. 2013). Amplitudes of Waves I at 90 dB SPL were evaluated.

### Histology and immunofluorescence (IF) staining

Mice were heavily anesthetized with ketamine and xylazine, and euthanized by cervical dislocation. Cochleae were fixed with 4% paraformaldehyde (PFA) overnight at 4 °C. After washed with PBS three times, parts of cochleae were decalcified in 10% EDTA for 48 h at 4 °C and then were washed with PBS. Samples were dehydrated in a gradient of 20%, and 30% sucrose (1 h each) at 4 °C. Subsequently, samples were embedded in optimal cutting temperature compound, and then were sliced at − 20 °C with a thickness of 10 μm using a Leica Cryastat (German). Cochlear frozen sections were stored at − 80 °C for IF staining. Remaining cochlea were decalcified for 2 h at room temperature. Cochleae were microdissected into three pieces: apical, middle, and basal turn.

For IF staining, frozen sections were washed with PBS three times (10 min each). Frozen sections and cochlear turns were permeabilized and blocked with PBS consisting of 5% goat serum and 0.3% TritonX-100 for 2 h, and incubated with primary antibodies overnight at 4 °C. The primary antibodies were applied as follows: mouse anti-4 hydroxynonenal (4-HNE, diluted 1:200; Abcam, USA), a biomarker of oxidative damage; mouse anti-8-hydroxy-2′-deoxyguanosine (8-OHdG, diluted 1:300; Abcam, USA), a marker of DNA oxidative damage; rabbit anti-Myosin VIIa (diluted 1:300; Proteus Biosciences, USA), a hallmark of cochlear hair cells; mouse anti-C-terminal binding protein 2 (CtBP2, diluted 1:300; BD Biosciences, USA), which is used to label the presynaptic ribbon; mouse anti-GFP (diluted 1:100, Santa Cruz Biotechnology, USA). Additionally, samples were immunostained with primary antibody to rabbit anti-phosphorylated AMP-activated protein kinase α (pAMPKα, diluted 1:50; Cell Signaling, USA) for 48 h at 4 °C. The following day, after washing with PBS three times, samples were incubated with at appropriate Alexa-conjugated secondary antibodies for 1 h at room temperature. All secondary antibodies were diluted at 1:300 in PBS. Samples were mounted with an anti-fluorescence quenching agent containing DAPI.

### Confocal microscopy and image analysis

All confocal images of three random fields per turn ('Apical', 'Middle', 'Basal') from each cochlea were captured using a Leica TCS SP8 laser microscope. Image analysis were performed as previously described with a minor modification (Kujawa and Liberman 2009; Sergeyenko et al. 2013).

To count OHCs and IHCs labeled with the anti-Myosin VIIa antibodies, images with a z-step-size of 2 μm were obtained with an oil immersion objective with 2 × digital zoom. The number of OHCs and IHCs in apical, middle, and basal turns was manually counted, respectively.

To count synapses labeled with anti-CtBP2, images with a z-step-size of 0.5 μm were obtained with 63×/1.40-NA oil objective with 2 × digital zoom. IHC synapses in apical, middle, and basal turns were quantified, respectively. And IHC synapses were divided by the total number of IHCs stained by anti-Myosin VIIa in per field under the microscope. If needed, 3D renderings were generated to avoid miscounting the number of synapses due to the image overlay process.

To quantify levels of 4-HNE and 8-OHdG, images with a z-step-size of 1 μm were obtained with oil immersion objective with 1.5 × digital zoom. The relative fluorescence intensities of 4-HNE and 8-OHdG were semi-quantified by measuring the average intensity from three random microscope fields in each cochlear.

To quantify the level of pAMPK per turn, images with a z-step-size of 1 μm were obtained with an oil immersion objective with 2 × digital zoom. The relative fluorescence intensity of pAMPK was semi-quantified by measuring the average intensity from three random microscope fields in each cochlear turn using ImageJ software (NIH, Bethesda, MD, USA), and normalized to controls.

### Mitochondrial ROS measurement

Mitochondrial ROS were determined by MitoSOX™ Red (Invitrogen, USA) according to the manufacturer's instructions. Briefly, after sacrificing the mice, part of the cochlear bone shell was quickly removed under a microscope. Because the cochlear shell is relatively hard in mice over 8 weeks old, it is difficult to completely remove it without decalcification or causing damage to hair cells. Thereafter, the samples were incubated with DMEM/F12 and 5 μM MitoSOX™ Red for 35 min at 37 °C and washed thrice with warm PBS, after which they were fixed with 4% PFA at 4 °C overnight. After again washing thrice with PBS, samples were decalcified in 10% EDTA for 1 h. Cochleae were then microdissected into apical, middle, and basal turns while being protected from light. The samples were then sealed with an anti-fluorescence quenching agent with DAPI and visualized using confocal microscopy. MitoSOX™ fluorescence intensity was quantified using ImageJ (NIH, Bethesda, MD, USA), and the data were normalized to normal cochlea controls.

### Mitochondrial isolation

A commercial tissue mitochondria isolation kit (Beyotime Biotechnology, China) was applied to extract mitochondria from mouse cochlea. Briefly, after washing with ice-cold PBS, cochlear tissue was cut into very small pieces, after which it was suspended in 10 volumes of PBS. Thereafter, the samples were incubated on ice for 3 min and centrifuged for 10–20 s. After removing the supernatant, the samples were added to 8 volumes of PBS containing 0.25 mg/mL trypsin and incubated on ice for 20 min. After removing the supernatant, samples were re-suspended in 2 volumes of mitochondrial extraction buffer to remove residual trypsin, and then samples were centrifuged for 10–20 s at 600×*g*. Subsequently, the supernatant was removed, the samples were added to 8 volumes of mitochondrial extraction buffer, and then homogenized 20–30 times. The samples were centrifuged at 600×*g* for 5 min. Next, the supernatant was transferred to a clean tube and centrifuged at 11,000×*g* for 10 min. The centrifuged deposit was the mitochondria.

### ATP and MMP measurement

According to the manufacturer's instructions, ATP and MMP levels were measured with a commercial ATP and JC-1assay kit (Beyotime, China), respectively. The levels of ATP were quantified with relative luminescent units using a luminometer. ATP results were shown as nmol/mg protein. MMP levels were defined as the ratio of optical density between JC-1 aggregates to JC-1 monomers.

### TUNEL assay

The apoptosis of hair cells was measured with a TUNEL assay kit (Beyotime, China). After being permeabilized and blocked, samples were incubated with rabbit anti-Myosin VIIa (diluted 1:300; Proteus Biosciences, USA) and mouse anti-GFP (diluted 1:100, Santa Cruz Biotechnology, USA) overnight at 4 °C. The next day, after washing with PBS, samples were incubated with secondary Alexa Fluor 647 goat anti-rabbit IgG (1:300, Invitrogen, USA) and TUNEL red solution at room temperature for 1 h. Slides were mounted and imaged by confocal microscopy. The apoptosis rate refers to the proportion of TUNEL^+^ cells in the number of IHCs in three random microscopic fields per turn in each sample.

### Western blot (WB)

Mitochondria were extracted from cochlear tissues as described above. Briefly, the mitochondria from mouse cochlea were isolated with a tissue mitochondria isolation kit (Beyotime, China) according to the manufacturer’s instructions. The protein from mouse cochlea tissues was extracted with strong RIPA lysis buffer containing PMSF (Sigma, USA) and protease inhibitor cocktail (Thermo Fisher Scientific, USA). Then, the protein was quantified. The protein was separated using 10% SDS-PAGE gels and then blotted onto PVDF membranes. After being blocked, the membranes were exposed to primary antibodies overnight at 4 °C: anti-UCP2 (diluted 1:1000; Abcam, USA); anti-superoxide dismutase 2 (SOD2, diluted 1:1000; Cell Signaling, USA); anti-AMPK (diluted 1:1000; Cell Signaling, USA); anti-pAMPK (diluted 1:1000; Cell Signaling, USA); β-actin (diluted 1:1000; Cell Signaling, USA); α-tubulin (diluted 1:1000; Cell Signaling, USA); and GAPDH (diluted 1:1000; Cell Signaling, USA). Following day, the membranes were incubated with appropriate secondary antibodies. The protein signals were measured by enhanced chemiluminescence, and quantified using the Fujifilm LAS 400 imaging system.

### Statistical analysis

SPSS (version 24.0, Chicago, IL, USA) was used for statistical analyses. Normality was analyzed with the Kolmogorov–Smirnov test. Data conforming to normal distribution are expressed as mean ± standard error of the mean (SEM). Comparisons between two groups were analyzed with a Student’s t test (two way); multiple comparisons were analyzed with the one-way analysis of variance (ANOVA) followed by the LSD’s post hoc test. A value of p < 0.05 was judged as significant. *p < 0.05; **p < 0.01.

## Results

### Mitochondrial dysfunction occurs in IHCs of ARHL

**Fig. 1:**
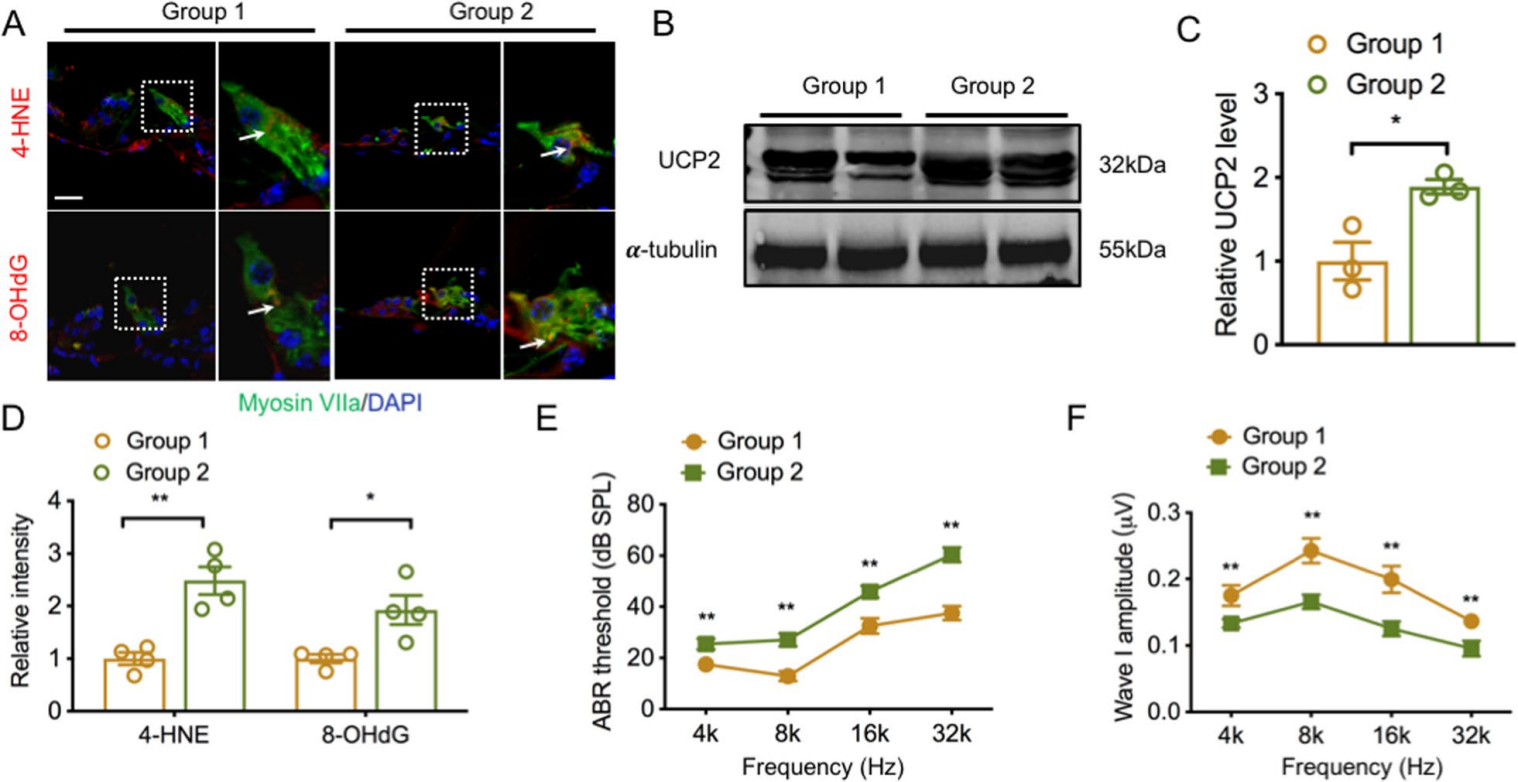
Oxidative stress occurs in IHCs of ARHL. **A** Representative IF staining of 4-HNE (red) and 8-OHdG (red) in the cochlea from different groups. IHCs and nuclei were stained with anti-Myosin VIIa (green) and DAPI (blue), respectively. Scale bar, 20 μm. **B** Representative western blots for UCP2 in the different groups. **C** Western blot analysis of the relative UCP2 expression (n = 3 independent experiments). **D** The relative fluorescence intensities of 4-HNE and 8-OHdG, respectively (n = 4 ears/group). **E** The mean ABR thresholds at four frequencies of 4, 8, 16 and 32 kHz (n = 12 ears/group). **F** The mean ABR wave I amplitudes at all tested frequencies (n = 12 ears/group). IHC, inner hair cell; ARHL, age-related hearing loss; UCP2, uncoupling protein 2. *p < 0.05, **p < 0.01

We first evaluated the role of oxidative damage in the cochlea by determining three hallmarks, 4-HNE, 8-OHdG, and MitoSOX. IF analysis displayed that 4-HNE and 8-OHdG were both located in the cytoplasm of IHCs (Fig. 1A). The fluorescence intensity of 4-HNE in IHCs in group 2 was much higher than that in group 1. Similarly, the 8-OHdG fluorescence intensity in group 2 was notably enhanced in comparison with that in group 1 (Fig. 1D). MitoSOX staining demonstrated a remarkably greater fluorescence intensity in group 2, compared with that in group 1, in both the apical, middle, and basal turns (Fig. 4), suggesting increased mitochondrial oxidative damage in the cochleae of 16w mice. Furthermore, WB analysis showed that UCP2 levels increased approximately 1.8-fold in the cochleae of group 2 mice, compared with those of group 1 mice (Fig. 1B, C).

To further investigate the role of UCP2 in ARHL, we evaluated cochlear function by testing hearing thresholds at different frequencies and ABR wave I amplitudes in 8w and 16w C57BL/6 J mice. The objective electrophysiological ABR test showed that the hearing thresholds of group 2 mice were remarkably higher than those of group 1 mice at frequencies ranging from 4 to 32 kHz (Fig. 1E), with the largest hearing threshold shift observed at 32 kHz. Additionally, the ABR wave I amplitudes at all measured frequencies in group 2 were much lower than those in group 1 (Fig. 1F). These data demonstrated that C57BL/6 J mice at age of 16w displayed representative hearing loss in ARHL. Collectively, these results suggest that mitochondrial oxidative damage is related to early-onset of ARHL, and UCP2 may play an important role in ARHL.

### UCP2 overexpression aggravates hearing loss in ARHL

**Fig. 2:**
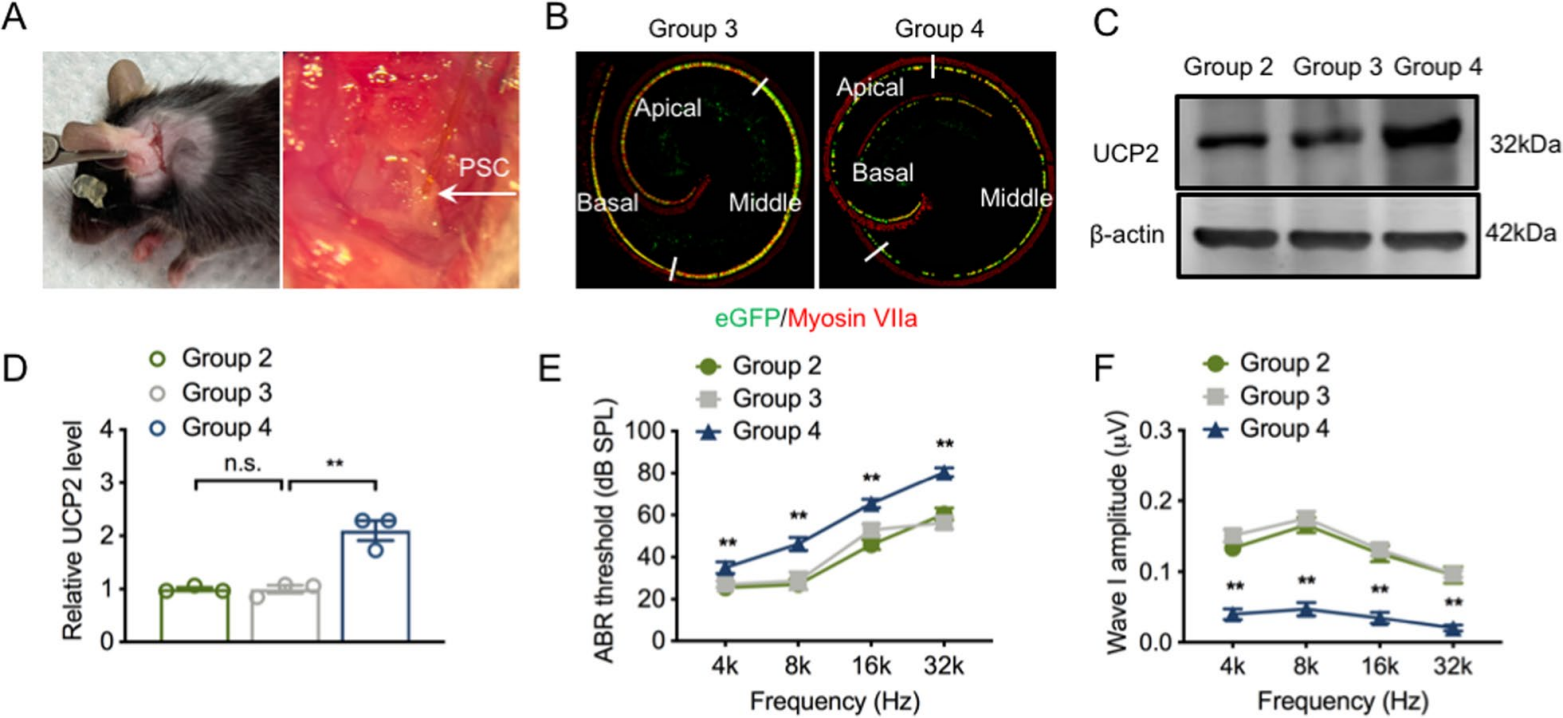
UCP2 overexpression notably aggravates hearing loss. **A** The AAV-ie vectors are infected through the PSC. **B** Representative merged images of cochlear basilar membrane in the different groups. IHCs were labeled with anti-Myosin VIIa (red). **C** Representative western blots for UCP2 protein (n = 3 independent experiments). **D** The relative expression of UCP2 protein. **E** The mean ABR thresholds at four tested frequencies (n = 12 ears/group). **F** The mean ABR wave I amplitudes at all tested frequencies (n = 12 ears/group). UCP2, uncoupling protein 2; IHCs, inner hair cells; PSC, posterior semicircular canal; ABR, auditory brainstem response; OHCs, outer hair cells. n.s., no significance, *p < 0.05, **p < 0.01

Since UCP2 can protect cells and tissues against oxidative damage, we used an AAV-ie vector to deliver UCP2 into the inner ear via the PSC to explore in detail the effect of UCP2 on the inner ear in early-onset of ARHL (Fig. 2A). The data displayed that cochlear IHCs were efficiently transfected by the AAV-ie vector (Fig. 2B). WB analysis showed that UCP2 levels were higher in group 4 than those in group 3 (Fig. 2C, D).

To further evaluate the role of UCP2 on hearing function, we analyzed hearing thresholds and wave I amplitudes in the different groups. As already shown in Fig. 1, the hearing results from group 2 were added for further comparison. No significant differences were observed in ABR hearing thresholds at all tested frequencies between group 2 and group 3 (Fig. 2E). ABR wave I amplitudes were also unchanged between the two groups at frequencies of 4–32 kHz (Fig. 2F). Interestingly, UCP2 overexpression did not improve hearing in 16w mice. In contrast, hearing thresholds at all four frequencies in group 4 significantly worsened compared with those in the group 3 (Fig. 2E). Moreover, UCP2 overexpression considerably decreased wave I amplitudes at all tested frequencies in the 16w mice (Fig. 2F). Collectively, these data suggest that UCP2 overexpression may have no protective role in ARHL, but instead exacerbates IHC and hearing losses in mice.

### UCP2 overexpression reduced the number of IHC synapses and IHCs in ARHL

**Fig. 3:**
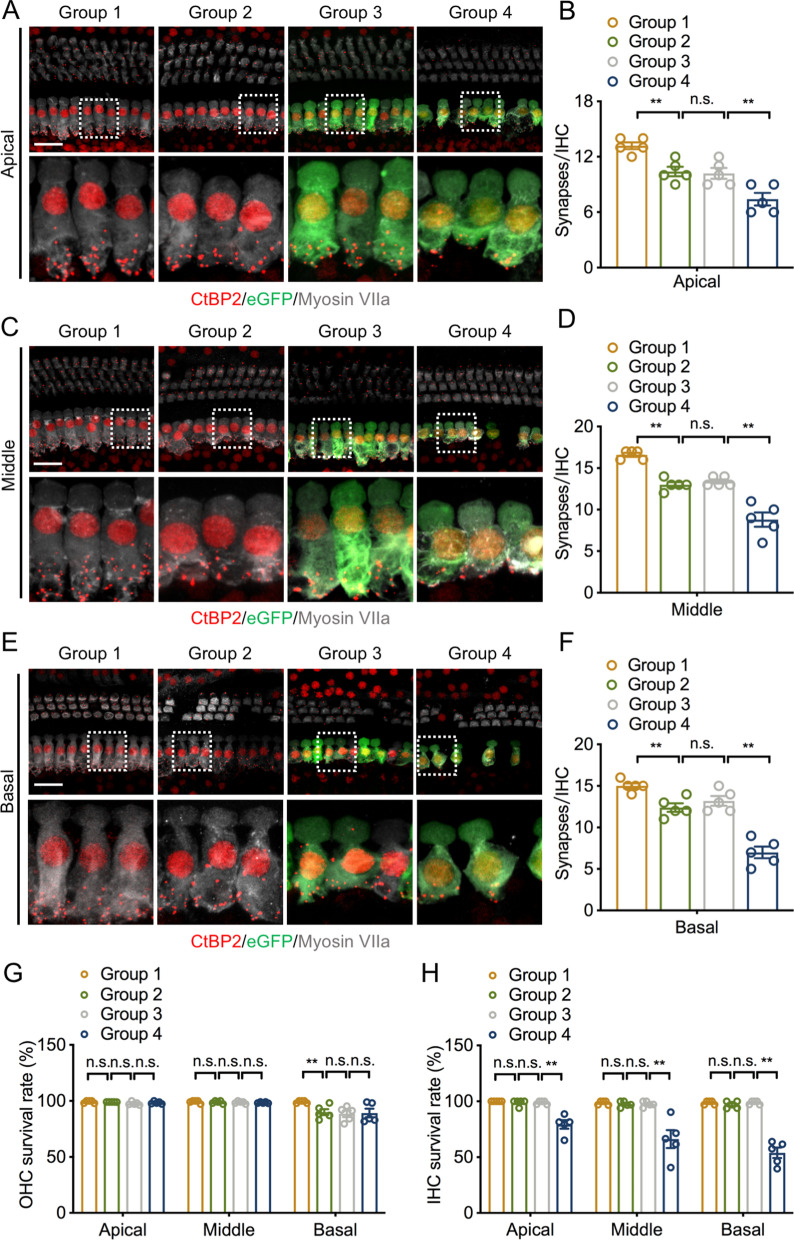
UCP2 overexpression decreases the number of IHC synapses and IHCs. **A**, **C** and **E** Representative images of IHC synapses (CtBP2, red) in the apical, middle and basal turns of the cochlea, respectively. IHCs and AAV-ie vector were labeled with anti-Myosin VIIa (grey) and anti-GFP (green), respectively. Scale bar, 20 μm. **B**, **D** and **E** Quantification the number of IHC synapses in the apical, middle and basal regions of the cochlea, respectively (n = 5 ears/group). **G**, **H** Percentage of survival OHCs and IHCs in the apical, middle and basal turns of the cochlea (n = 5 ears/group). *UCP2* uncoupling protein 2, *IHC* inner hair cell, *n.s.* no significance, *p < 0.05, **p < 0.01

Ribbon synapses play an important role in maintaining normal hearing; therefore, we next investigated the effect of UCP2 overexpression on IHC synapses. Postsynaptic biomarkers, including glutamate receptors and proteins related to postsynaptic density, remained unstained following IF staining; therefore, only presynaptic ribbons in the IHC regions were counted. A significant decrease in the number of IHC synapses in group 2 was observed in both the apical, middle, and basal turns compared with those in group 1 (Fig. 3). Mean counts from 16w mice exhibited ~ 10 synapses/IHC in the apical turn, ~ 13 synapses/IHC in the middle turn, and ~ 12 synapses/IHC in the basal turn. There was no noticeable difference in the number of IHC synapses in all turns between group 2 and group 3. Moreover, the data showed that overexpressing UCP2 further aggravated the loss of IHC synapses in all cochlear turns. In the apical turn, the number of synapses declined to ~ 7/IHC after UCP2 overexpression (Fig. 3B). Furthermore, IHC synaptic counts in the middle turn in mice transfected with AAV-ie-UCP2 were much less than those in AAV-ie-GFP mice (Fig. 3C, D). Additionally, mice with overexpressed UCP2 showed significant IHC synapse loss in the basal turn (Fig. 3E, F).

Next, we explored the effect of UCP2 overexpression on IHCs. Consistent with hearing data at high frequency (32 kHz), 16w mice showed remarkable OHC loss in the basal turn (Fig. 3G), indicating that OHCs in the basal turn of the cochlea are much more susceptible to damage in aging mice. As expected, the number of IHCs and OHCs in group 3 was consistent with that in the group 2. However, the 16w mice overexpressing UCP2 showed a significantly reduced number of IHCs in the apical, middle, and basal turns of the cochlea compared with that in the group 3 (Fig. 3H). Overall, these data suggest that the cochlea in ARHL exhibits age-related IHC synapse loss, and UCP2 overexpression in the inner ear may negatively modulate the number of IHC synapses and IHCs in ARHL.

### UCP2 overexpression aggravates mitochondrial dysfunction and increases AMPK activation

**Fig. 4:**
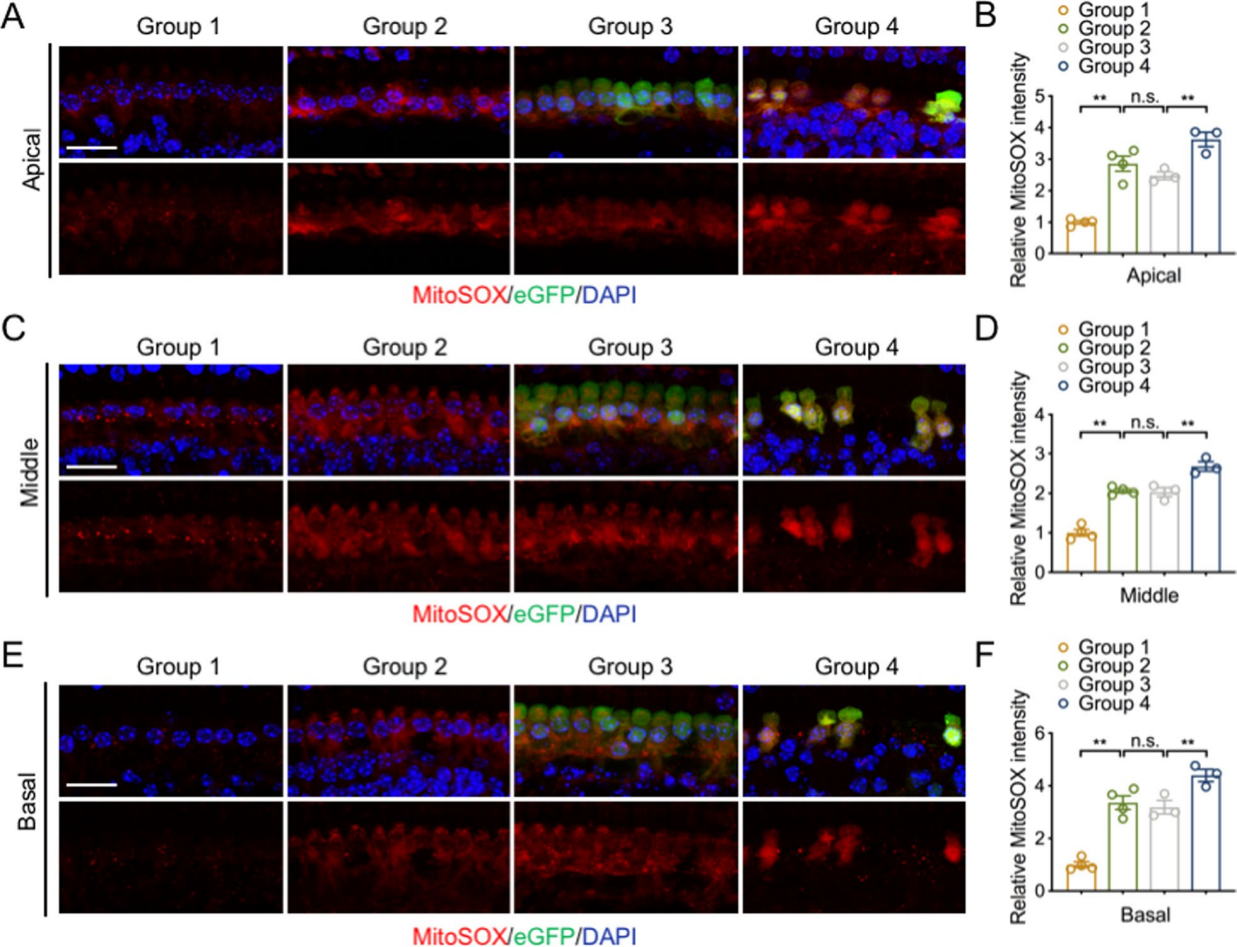
UCP2 overexpression increases mitochondrial oxidative stress. **A**, **C** and **E** Representative IF images of mitoSOX (red) in the apical, middle and basal turns in the different groups. Nuclei were labeled with DAPI (blue). Scale bar, 20 μm. **B**, **D** and **F** The relative fluorescence intensity of mitoSOX in the apical, middle and basal turns in the different groups (n = 3–4 ears/group). *UCP2* uncoupling protein 2, *n.s.* no significance, **p < 0.01

**Fig. 5:**
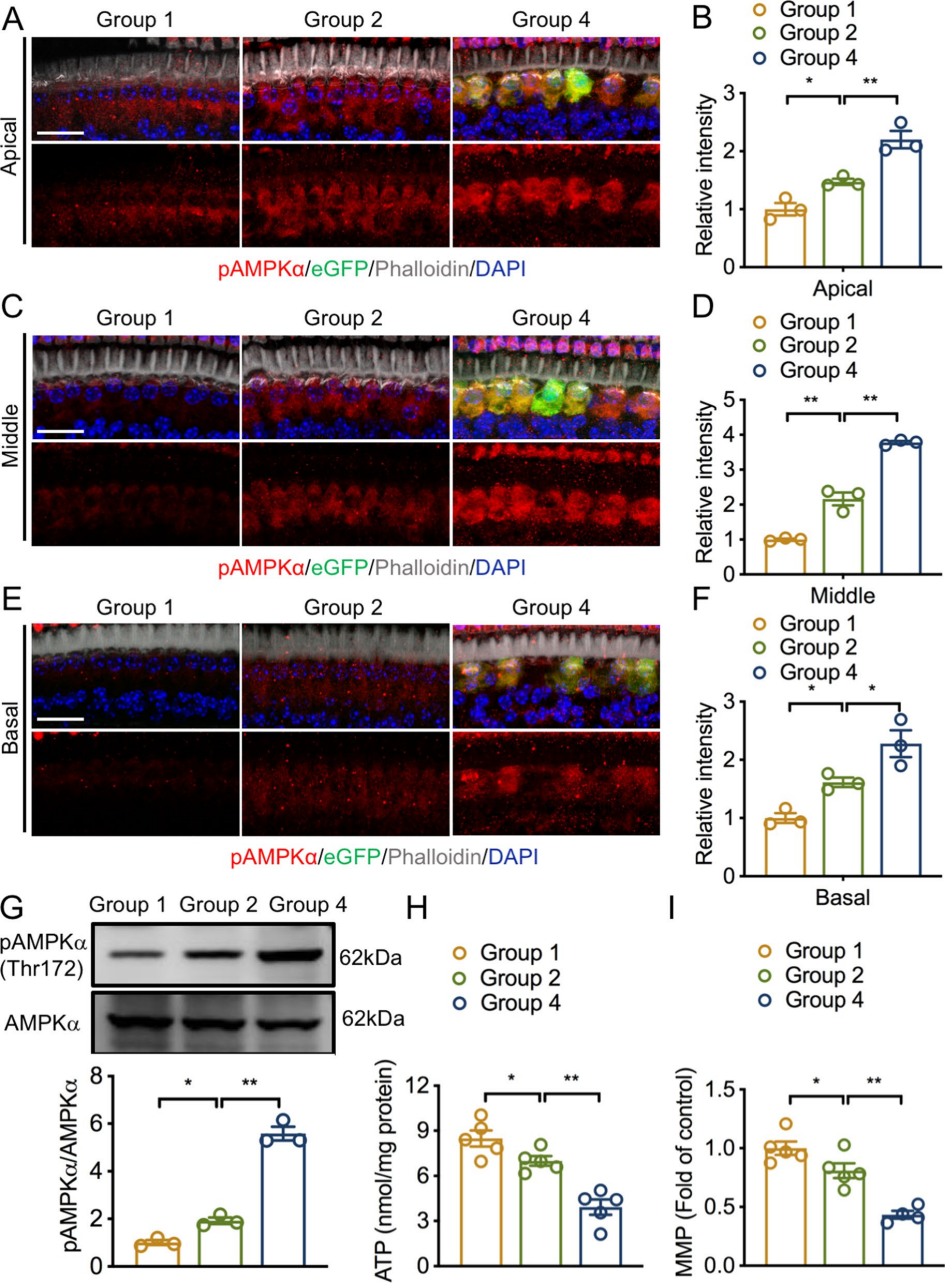
UCP2 overexpression reduces ATP and MMP levels and promotes AMPK activation. **A**, **C** and **E** Representative IF images of AMPKα (red) in the apical, middle and basal turns. AAV-ie vector and nuclei were labeled with anti-GFP (green) and DAPI (blue), respectively. Scale bar, 20 μm. **B**, **D** and **F** The relative fluorescence intensity of pAMPKα in the apical, middle and basal turns (n = 3 ears/group). **G** Western blots and analysis for pAMPKα and AMPKα in the different groups (n = 3 independent experiments). **H** The ATP levels measured by a commercial ATP assay kit (n = 5 independent experiments). **I** The MMP levels measured by a JC-1 assay kit (n = 4–5 independent experiments). *UCP2* uncoupling protein 2, *ATP* adenosine triphosphate, *AMPK* AMP-activated protein kinase α, *pAMPK* phosphorylated AMPK, *MMP* mitochondrial membrane potential, *n.s.* no significance, *p < 0.05, **p < 0.01

To identify the underlying mechanism of IHC loss upon UCP2 overexpression, we explored whether mitochondrial dysfunction resulted in increased sensitivity to IHC loss. MitoSOX staining showed no statistical difference in relative fluorescence intensity between groups 2 and 3, in neither the apical, middle, nor basal turn. However, compared with that in group 3, MitoSOX fluorescence intensity was notably increased upon UCP2 overexpression in the apical, middle, and basal turns (Fig. 4). Previous studies have suggested that low ATP levels can activate signaling and contribute to cell death (Shang et al. 2009). As mentioned above, AAVie vector transfection did not impact on hearing function or IHC morphology in mice. Thus, 16w mice were used for sequential mechanisms research. The data showed that ATP and MMP levels in group 2 were much lower than those in the group 1, and UCP2 overexpression significantly reduced ATP and MMP levels (Fig. 5H, I), indicating that UCP2 overexpression may inhibit ATP production in the cochlea.

The AMPK, an energy-sensing kinase, is a critical molecule in the modulation of cellular energy metabolism and can be activated by decreased ATP levels (Herzig and Shaw 2018). We thus investigated whether AMPK was activated in the IHCs of mice with UCP2 overexpression. Activated AMPKα (Thr172), observed as red fluorescence, was located in the cytosol, as displayed by red fluorescence (Fig. 5). IF imaging analysis indicated that the relative intensity of pAMPKα in the apical, middle, and basal turns of IHCs in group 2 was markedly increased relative to that in group 1. Strikingly, overexpressing UCP2 significantly enhanced the relative intensity of pAMPKα in all cochlear turns, as shown by IF staining (Fig. 5A–F). Consistent with IF observations, WB analysis also showed that pAMPKα levels were significantly elevated in group 2 compared with those in the group 1. Moreover, a further increase in the level of pAMPKα was seen in the group 4, compared with that in group 2 (Fig. 5G). Collectively, these data suggest that UCP2 overexpression-triggered IHC loss may be associated with ATP and MMP reduction and AMPK activation.

### UCP2 overexpression promotes hair cell apoptosis in ARHL

**Fig. 6:**
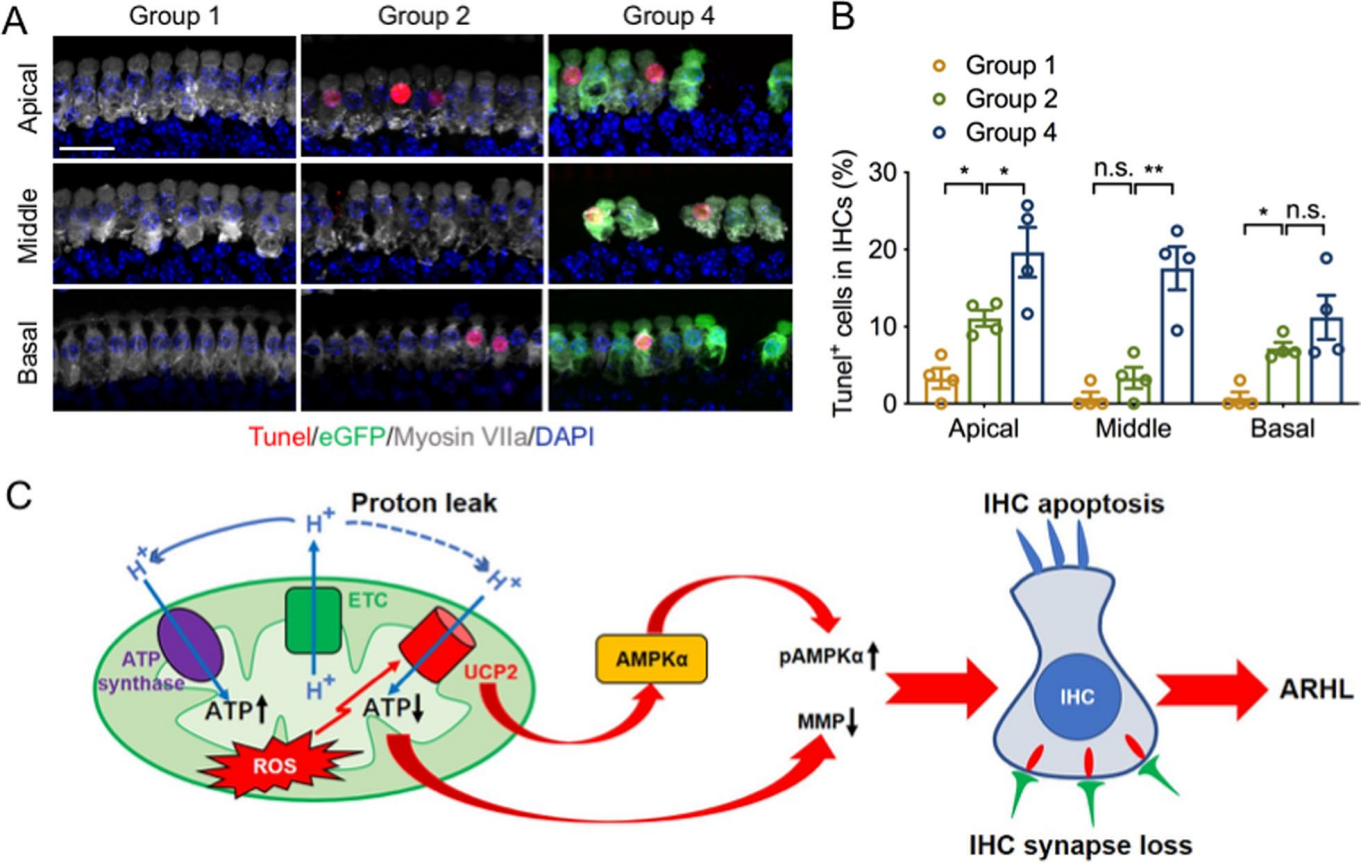
UCP2 overexpression promotes IHC apoptosis in ARHL. **A** Representative IF images of TUNEL^+^ DAPI^+^ cells in the different groups. AAV-ie vector and IHCs were labeled with anti-GFP (green) and anti-Myosin VII (grey), respectively. Scale bar, 20 μm. **B** The ratio of IHC apoptosis in different groups (n = 4 ears/group). **C** Schematic model explaining the role of UCP2 based on our results in ARHL. *UCP2* uncoupling protein 2, *IHC* inner hair cell, *ARHL* age-related hearing loss, *ATP* adenosine triphosphate, *MMP* mitochondrial membrane potential, *pAMPK* phosphorylated AMP-activated protein kinase, *n.s.* no significance, *p < 0.05, **p < 0.01

We next examined the role of UCP2 overexpression in cell apoptosis in ARHL as evaluated by a TUNEL assay. In the apical turn, 16w mice showed a higher percentage of TUNEL^+^ IHCs compared with those in 8w mice; with UCP2 overexpression further elevating the ratio of TUNEL^+^ IHCs. In the middle turn, no statistical differences in the ratio of TUNEL^+^ IHCs were observed between group 2 and group 1; however, a higher proportion of TUNEL^+^ cells was found in the IHCs of UCP2 overexpressed mice compared with those in group 2 (Fig. 6A, B). In the basal turn, the percentage of TUNEL^+^ IHCs in group 2 was elevated compared with that in group 1; however, UCP2 overexpression did not lead to an increase in the proportion of TUNEL^+^ IHCs compared with that in 16w mice. Nevertheless, these data demonstrate that UCP2 overexpression in IHCs may have a proapoptotic role in early-onset of ARHL.

## Discussion

Mitochondrial dysfunction is particularly associated with aging and age-related diseases (Amorim et al. 2022). In this study, we hypothesized that UCP2 overexpression could exacerbate the damage to IHCs and promote hearing loss by regulating cellular apoptosis in ARHL. Consistent with a previous study, we found that UCP2 and ROS were increased in the cochleae in ARHL (Du et al. 2020). UCP2 promotes the 'proton leak' to reduce the MMP, inhibiting ATP synthesis, and decreasing ROS generation. Overexpressing UCP2 accelerated IHC and IHC synapse losses, decreased levels of ATP and MMP levels, and increased ROS production, indicating that overexpressing UCP2 further leads to impairment of IHC and mitochondrial function and disruption of energy homeostasis, which could cause severe hearing loss induced by UCP2 targeting. Alternatively, our data also showed that UCP2 overexpression promoted the activation of AMPKα Thr172 phosphorylation, which may be mediated by energy imbalance. Therefore, our data indicate that UCP2 overexpression accelerates hearing loss and related pathophysiological changes in ARHL.

During the past few decades, numerous studies have proposed that oxidative damage plays an essential role in ARHL (Kim et al. 2019; Rousset et al. 2020; Someya et al. 2009). We also observed excessive ROS formation in the cochleae of C57BL/6 J mice, one of the most widely applied rodent models for studying aging and age-related diseases. Accumulated ROS cause mitochondrial components injury and mitochondrial dysfunction, which contribute to cytotoxicity and the activation of the mitochondrial apoptotic pathway and, ultimately, to ARHL (Bermudez-Munoz et al. 2020; Rousset et al. 2020). Thus, it is particularly important to scavenge excessive ROS, reduce their cytotoxicity, protect IHCs against oxidative damage, and maintain redox homeostasis. UCPs, including UCP1, UCP2, and UCP3, play vital roles in regulating ROS and cellular function (Krauss et al. 2005). Our observations in the present study are in consistent with previous studies showing that UCP2 expression is upregulated in the cochleae in ARHL (Du et al. 2019, 2014; Park et al. 2020). UCP2, a homologue of UCP1, is a classical uncoupling protein in the inner mitochondrial membrane (Krauss et al. 2005), that is involved in reducing ROS generation, attenuating oxidative stress, and preventing ROS-induced apoptosis, and plays a protective role in hypothalamic neurons, respiratory epithelial cells and myocardial cells (Andrews et al. 2008; Teshima et al. 2003; Wang et al. 2018). However, in the present study, mice overexpressing UCP2 has significantly higher hearing threshold, reduced number of IHCs and IHC synapses, increased oxidative stress, and much lower ATP concentrations compared with those in controls, indicating that UCP2 exacerbated the degeneration of IHCs in ARHL possibly through energy metabolic disturbance.

The auditory sense is contingent on fast and precise synaptic transmission at cochlear hair cell synapses (Keen and Hudspeth 2006). Synaptic activity expends considerable energy, provided as ATP generated via glycolysis and OXPHOS (Li and Sheng 2022). Some studies demonstrated that UCP2 has a neuroprotective role in several neuronal damage models (Andrews et al. 2005; Islam et al. 2012; Paradis et al. 2003). Although UCP2 overexpression resulted in insufficient ATP generation per mitochondrion, the number of mitochondria was elevated in the hippocampus, ultimately leading to an overall increase in ATP levels, thus indicating that the protective effect of UCP2 on neurons in the hippocampus of epileptic mice might be mediated by mitochondrial proliferation (Diano et al. 2003). Mitochondrial biogenesis, quantity, mass, and position are crucial for maintaining synaptic transmission, synaptic plasticity, and synaptic homeostasis (Devine and Kittler 2018). Yamada et al. reported that adenovirus-UCP2 transfection of PC12h cells induced decreased dopamine secretion and ATP levels in vitro (Yamada et al. 2003), which might be relevant to acute overexpression of UCP2 over a short period. Enhanced uncoupling might damage mitochondrial function, causing a period of ATP reduction, differing from chronic neuronal UCP2 function (Andrews et al. 2005). Consistent with these findings, our data showed that the UCP2 overexpression caused ATP depletion. However, we speculate that ATP depletion may be due to UCP2-mediated excessive uncoupling of mitochondrial OXPHOS, resulting in a dramatic decrease in ATP levels and mitochondrial metabolic changes. Alternatively, ATP depletion is possible because the effect of UCP2 on mitochondrial function has tissue-specific sensitivities. For example, UCP2 upregulation in islets of ob/ob mice caused reduced ATP synthesis and attenuated insulin release, ultimately developing obesity and type 2 diabetes mellitus (Zhang et al. 2001). In contrast, increased UCP2 contributes to reducing obesity because UCP2 upregulation augments energy expenditure in adipocytes and skeletal muscle (Esterbauer et al. 2001; O’Rahilly 2001).

There is a feedback loop between ROS and UCP2-dependent uncoupling. Our results indicated that UCP2 levels were higher in the 16w mice than 8w mice. Although the mechanism whereby ROS regulates UCP2 is not fully understood, increased ROS activate the proton leak of UCP2 and upregulates its expression (Brand and Esteves 2005). According to the 'uncoupling to survive', UCP2 dissipates the proton gradient by reducing MMP, consequently decreasing ROS generation (Brand 2000). When targeted expression of human UCP2 to neurons in adult flies, the flies showed an increase in uncoupled respiration, a reduction in ROS generation and oxidative damage, and an extension of life span, suggesting that mitochondrial uncoupling diminishes age-related oxidative damage (Fridell et al. 2005). The potential role of UCP2 in longevity by regulating ROS production is supported by several further studies (Andrews and Horvath 2009; Andrews et al. 2005; Brand and Esteves 2005). However, in the present study, UCP2 overexpression resulted in mitochondrial ROS production and exacerbated oxidative damage. UCP2 has a dual role in regulating the ATP synthase and modulating redox homeostasis (Krauss et al. 2005; Kumar et al. 2022). Upregulation of UCP2 within a certain range, or compensatory increase, is protective and causes mild uncoupling, thus reducing ROS production and mitigating oxidative damage; whereby UCP2 overexpression causes excessive mitochondrial uncoupling, which is harmful. Our results revealed that UCP2 overexpression led to decreased ATP and MMP levels. As the normal physiological function of cochlear hair cells cannot be maintained due to reduced ATP, mitochondria are required to accelerate ATP production to achieve normal cellular homeostasis, resulting in increased proton leak in OXPHOS, which leads to increased ROS levels, further disrupting mitochondrial function and causing hair cell loss, and consequently, hearing loss.

As an uncoupling protein, UCP2 has been indicated to impact many crucial processes in cellular function (Andrews et al. 2008; Paradis et al. 2003; O’Rahilly 2001; Teshima et al. 2003). Our data showed that UCP2 overexpression promoted the activation of AMPK, an evolutionarily conserved serine/threonine kinase and heterotrimeric complex made of α, β, and γ subunits that acts as an energy-sensing switch to modulate cellular metabolic homeostasis (Herzig and Shaw 2018). AMPK can be activated by inadequate ATP provision, increased energy expenditure, and excessive ROS (Trefts and Shaw 2021). In the present study, compared with those from 8w mice, IHCs from 16w mice showed increased ROS levels and AMPK activation. Our data further demonstrated that ROS is a non-canonical trigger of AMPK activation. Similarly, in noise-induced hearing loss (NIHL), oxidative stress induced by ROS is considered an activator of AMPK (Hill et al. 2016; Wu et al. 2020). Our results showed that UCP2 overexpression elevated the phosphorylation of AMPK, suggesting that AMPK was activated by reduced ATP production. In D-galactose-induced ARHL, AMPK activation by metformin alleviated hearing loss, suppressed cell apoptosis, and mitigated neurodegeneration, which were attributable to decreased ROS levels (Cai et al. 2020). However, our results showed that AMPK activation induced severe IHC damage and apoptosis. A recent study assessed the effect of AMPK inhibition on hair cell death in NIHL (Hill et al. 2016). The data exhibited that AMPK downregulation played a protective role in NIHL, where reduced OHC and IHC synaptic losses and hearing loss were observed. Moreover, Shang et al. showed UCP2 overexpression led to declined ATP generation and AMPK activation, and sustained AMPK activation caused c-Jun N-terminal kinase activation, resulting in hepatocyte apoptosis. Furthermore, they suggested that exogenous UCP2 overexpression expression decreased the energy coupling effectiveness for OXPHOS and disrupted mitochondrial function, resulting in elevated susceptibility to liver damage (Shang et al. 2009). Alternatively, we speculate that the role of AMPK in ARHL may be connected with energy metabolism. Overexpression of UCP2 in IHCs in ARHL leads to substantially decreased ATP levels, which causes AMPK activation that restores energy homeostasis. When uncoupling surpasses the capacity of mitochondria to generate ATP, these responses are unable to attenuate energy stress and metabolic disorder, ultimately inducing in irreversible cell death (Green et al. 2014).

UCP2 expression affects the mitochondrial respiratory chain which is essential to cellular homeostasis. Whether UCP2 overexpression-mediated metabolic abnormality is the immediate cause of hair cell and hearing losses requires further investigated. Alternatively, several studies have explored the mechanoregulation of UCP2 inhibition or knock-out in several cells, such as pancreatic islet cells (Zhang et al. 2006), and proximal tubular cells (Ke et al. 2020). Our data suggest that UCP2 overexpression might cause mitochondrial dysfunction and cell apoptosis in cochlear hair cells during aging. However, to further understand the role of UCP2, it is necessary to explore the effects of UCP2 on the inner ear in UCP2-knockout or knockdown mice in a future study.

## Conclusions

In the present study, our results suggest that UCP2 overexpression exacerbated the disruption of IHCs and hearing in ARHL. Furthermore, it may be associated with energy metabolism imbalance induced by ATP reduction in responses to exogenous UCP2 overexpression, eventually leading to IHC death. These data indicate the harmful role of UCP2 in ARHL and how UCP2 may negatively regulate IHC function and hearing in ARHL.

## Data Availability

The data used in the present study are available from the corresponding author on reasonable request.

## Abbreviations

UCP2: Uncoupling protein 2
ROS: Reactive oxygen species
ARHL: Age-related hearing loss
AAVie: Adeno-associated virus-inner ear
IHC: Inner hair cell
AMPKα: AMP-activated protein kinase α
OHCs: Outer hair cells
SGNs: Spiral ganglion neurons
ATP: Adenosine triphosphate
OXPHOS: Oxidative phosphorylation
MMP: Mitochondrial membrane potential
4-HNE: 4-Hydroxynonenal
8-OHdG: 8-Hydroxy-2′-deoxyguanosine
CtBP2: C-terminal binding protein 2
